# Surface Characterization and Physiochemical Evaluation of P(3HB-*co*-4HB)-Collagen Peptide Scaffolds with Silver Sulfadiazine as Antimicrobial Agent for Potential Infection-Resistance Biomaterial

**DOI:** 10.3390/polym13152454

**Published:** 2021-07-26

**Authors:** Sevakumaran Vigneswari, Tana Poorani Gurusamy, Wan M. Khairul, Abdul Khalil H.P.S., Seeram Ramakrishna, Al-Ashraf Abdullah Amirul

**Affiliations:** 1 Faculty of Science and Marine Environment, Universiti Malaysia Terengganu, Kuala Terengganu 21030, Terengganu, Malaysia; vicky@umt.edu.my (S.V.); wmkhairul@umt.edu.my (W.M.K.); 2 School of Biological Sciences, Universiti Sains Malaysia, Gelugor 11800, Penang, Malaysia; purani_guru@yahoo.com; 3 School of Industrial Technology, Universiti Sains Malaysia, Gelugor 11800, Penang, Malaysia; akhalilhps@gmail.com; 4 Center for Nanofibers and Nanotechnology, Department of Mechanical Engineering, National University of Singapore, Singapore 117581, Singapore; seeram@nus.edu.sg; 5 Centre for Chemical Biology, Universiti Sains Malaysia, Bayan Lepas 11900, Penang, Malaysia; 6 Malaysian Institute of Pharmaceuticals and Nutraceuticals, NIBM, Gelugor 11700, Penang, Malaysia

**Keywords:** P(3HB-*co*-4HB), silver sulfadiazine, collagen peptide, infection-resistance scaffolds

## Abstract

Poly(3-hydroxybutyrate-*co*-4-hydroxybutyrate) [P(3HB-*co*-4HB)] is a bacterial derived biopolymer widely known for its unique physical and mechanical properties to be used in biomedical application. In this study, antimicrobial agent silver sulfadiazine (SSD) coat/collagen peptide coat-P(3HB-*co*-4HB) (SCCC) and SSD blend/collagen peptide coat-P(3HB-*co*-4HB) scaffolds (SBCC) were fabricated using a green salt leaching technique combined with freeze-drying. This was then followed by the incorporation of collagen peptides at various concentrations (2.5–12.5 wt.%) to P(3HB-*co*-4HB) using collagen-coating. As a result, two types of P(3HB-*co*-4HB) scaffolds were fabricated, including SCCC and SBCC scaffolds. The increasing concentrations of collagen peptides from 2.5 wt.% to 12.5 wt.% exhibited a decline in their porosity. The wettability and hydrophilicity increased as the concentration of collagen peptides in the scaffolds increased. In terms of the cytotoxic results, MTS assay demonstrated the L929 fibroblast scaffolds adhered well to the fabricated scaffolds. The 10 wt.% collagen peptides coated SCCC and SBCC scaffolds displayed highest cell proliferation rate. The antimicrobial analysis of the fabricated scaffolds exhibited 100% inhibition towards various pathogenic microorganisms. However, the SCCC scaffold exhibited 100% inhibition between 12 and 24 h, but the SBCC scaffolds with SSD impregnated in the scaffold had controlled release of the antimicrobial agent. Thus, this study will elucidate the surface interface-cell interactions of the SSD-P(3HB-*co*-4HB)-collagen peptide scaffolds and controlled release of SSD, antimicrobial agent.

## 1. Introduction

Biomaterial scaffolds are materials which have been engineered to interact with our biological system in providing three-dimensional structure and mimicking an extracellular matrix (ECM). Therefore, it is crucial to design biologically active scaffolds with well interconnected configuration and surface chemistry to enhance the cellular interactions on the scaffold interface [1,2]. The scaffold interface would enhance and facilitate the cell infiltration, proliferation and differentiation of cell lines, and eventually contribute to the tissue regeneration. 

Polyhydroxyalkonates (PHAs) are insoluble granules accumulated in cell cytoplasm as carbon and energy storage compounds under stress conditions [3,4,5]. PHAs are a biodegradable thermoplastic which exhibit similar thermo-mechanical properties to synthetic polymers [6]. Among the variety of PHAs, copolymer P(3HB-*co*-4HB) is widely used in biomedical applications due to the non-toxic biodegradation products, wide range of physical and mechanical properties, non-carcinogenic effects and biocompatibility [7]. It possesses exceptional properties for medical and pharmaceutical fields [8,9]. Moreover, P(3HB-*co*-4HB) has Food and Drug Administration (FDA) clearance for clinical usages among all the other PHAs available [3]. The P(3HB-*co*-4HB) was biosynthesized by bacterium *Cupriavidus necator* (formally *Ralstonia eutropha*) from structurally related sources such as 4-hydroxybutyric acid (4HBA), 4-chlorobutyric and γ-butyrolactone [7].

However, P(3HB-*co*-4HB) lacks active functional sites for cell attachment which limits the applications for regenerative medicine. Many studies have been carried out in this direction to overcome this limitation. Therefore, surface modification is carried out by incorporating natural polymers, such as collagen, gelatin, pullulan and chitosan, in enhancing the hydrophilicity of the scaffolds [10]. Nevertheless, the desirability and wide applicability of collagen is often attributed to its abundance in the human body as the key structural fibrous protein of the ECM [11]. Hence, collagen peptide was used as the biomolecules to enhance the hydrophilicity of the scaffolds fabricated in our study. Collagen peptide is a biomolecule which not only has the ability to improve the hydrophilicity of the scaffold but has the natural ability to interact with host cells [12,13].

Biomaterial scaffold-affiliated microbial infections are an emerging threat in clinical practices, which cause serious infection and impact healing. Therefore, designing scaffolds with antimicrobial efficacy have extensively gained priority in resolving biomaterial-associated infections [14]. Silver sulfadiazine (SSD) is an antibacterial agent that exhibits broad-spectrum antibacterial activity against Gram-positive and Gram-negative bacteria, as well as fungi, even at very low concentrations [15,16,17]. SSD is a much preferred antibacterial agent of choice due to the ability of SSD to reduce early infections at low concentration. However, currently available formulations of antimicrobial agents lack the ability to control the release of antimicrobial properties [18,19]. There are many scaffolds developed with antimicrobial properties and Table 1 lists common examples of antimicrobial biopolymer incorporated with SSD.

Following the aforementioned background, in the present work, the surface architecture of P(3HB-*co*-4HB) was enhanced by incorporating collagen peptides and silver sulfadiazine (SSD) as the antimicrobial mechanism agent. Two different scaffolds, namely SSD coat/collagen peptide coat-P(3HB-*co*-4HB) [SSCC] and SSD blend/collagen peptides coat-P(3HB-*co*-4HB) [SBCC] scaffold, were fabricated by the combination of salt leaching and freeze-drying techniques which are low cost and apply green technology to fabricate the scaffolds. The study provides evidence for increased hydrophilicity due to the incorporation of collagen peptide. This elucidates surface interface-cell interactions of the modified P(3HB-*co*-4HB) scaffolds and release mechanism of the antimicrobial agent from the scaffolds, thus driving the research effort forward for emerging infection-resisting biomaterials in tissue engineering and regenerative medicine in the future. 

## 2. Materials and Methods

### 2.1. Biosynthesis of P(3HB-co-95 mol% 4HB) Copolymer

The bacteria strains used in this study were *Cupriavidus malaysiensis* USMAA1020 transformant harbouring additional PHA synthase gene from *Cupriavidus malaysiensis* USMAA2–4 to produce P(3HB-*co*-95 mol% 4HB) copolymer. The biosynthesis was carried out as previously described [23]. A preculture of 5% (*v/v*) of the working volume was transferred into 20 L fermenter (Biostat^®^ C plus, Sartorius Stedim, German) containing mineral salts medium (MSM) with carbon precursors (1,4-butanediol and 1,6-hexanediol in the 1:5 ratio). The fermentation was carried out at 30 °C with an agitation speed of 200 rpm, the aeration rate of 1 vvm and controlled pH of 7 for 108 h. Sampling was done at intervals of every 12 h. The composition of PHA produced was determined by gas chromatography (GC) using Shimadzu Gas Chromatography GC-2014 according to methods previously described [24]. Endotoxin removal was carried out on extracted P(3HB-*co*-95 mol% 4HB) copolymer as previously described. The extracted polymer was characterized based on the molecular weight using Shimadzu GPC-2014 and tensile test using tensile testing machine (GoTech Al-3000, Shimadzu, Japan) [24].

### 2.2. Surface Functionalization of SSD/Collagen Peptide-P(3HB-co-4HB) Scaffolds

Surface functionalization of P(3HB-*co*-4HB) was carried out by salt leaching and solvent casting technique followed by freeze-drying method. Briefly, P(3HB-*co*-4HB) copolymer was dissolved in chloroform (5.5% w/v) and sodium bicarbonate (NaHCO_3_) particles sieved with known mesh sizes (200 µm) were added as porogen with mass ratio of salt:polymer at 6:1. The resulting polymer matrix was washed with deionized water to leach out the porogens. The scaffolds were freeze-dried for 24 h and later vacuum-dried for 48 h (BINDER GmbH, Tuttlingen, Germany) to remove any remaining solvent.

There were two types of scaffolds prepared using the various functionalization combination methods by incorporating different concentration of collagen peptide (2.5 wt.%, 5 wt.%, 7.5 wt.%, 10 wt.%, 12.5 wt.%) and 0.04% (w/v) of SSD. Collagen peptide powder from Tilapia fish skin with high purity (95%) and molecular weight of less than 3000 Da was used (Hainan Zhongxin Chemical Co. Ltd., Haikou, China).

The SSD coat/collagen peptide coat-P(3HB-*co*-4HB) scaffold (SCCC) was prepared by coating different concentration of collagen peptide in the silver (I) sulfadiazine (Sigma Aldrich) dispersed in hydrochloric acid solution (1.0 mM, pH 3.0).

The preparation of SSD blend/collagen peptide coat-P(3HB-*co*-4HB) scaffold (SBCC) was prepared with SSD added into the dissolved P(3HB-*co*-4HB) with NaHCO_3_ porogen and then solvent cast, as mentioned above.

Cross-linking was carried out using GA vapor-phase technique where the scaffolds were placed in an airtight desiccator containing 25% aqueous GA solution heated to 100 °C. Subsequently, the samples were washed for 24 h to remove GA, and then dried in vacuum for 24 h [8,25]. The scaffolds will be known as SCCC and SBCC from here on. 

### 2.3. Characterization of Scaffolds

The functional group present in the scaffolds fabricated were determined and analyzed using FTIR-ATR spectrophotometer (Model RX1, PerkinElmer, Buckinghamshire, UK). The spectra of the samples were obtained in the range of wave number between 650 cm^−1^ and 4000 cm^−1^. The spectrum of the FTIR was recorded in transmittance mode as function of wave number and the results were computed after 4 automated scans [24]. The surface morphology of the scaffolds coated with gold were mounted on aluminium stump and was observed using scanning electron microscopy (SEM) (Leo Supra 50 VP Field Mission SEM, Carl-Ziess SMT, Oberkochen, Germany). The scaffolds were cut into 1 cm × 1 cm. The dry weight before immersion (*m_o_*) was used as the initial weight of the scaffolds. The scaffolds were immersed in distilled water for 24 h. In order to obtain the wet weights (*m_f_*), the immersed scaffolds were removed from the solution, gently wiped with absorbent paper and air-died for 15 s before weighing. Water uptake was calculated using the formula below:

(1) $$Water\;uptake=\left(m_{o}-m_{f}\right)\;/\;m_{o}\times 100\%$$

The contact angle of the fabricated scaffolds was conducted by using sessile drop method (KSV CM200 Contact Angle) to determine their wettability properties. The scaffolds were cut into 1 cm × 1 cm pieces. The scaffolds were placed on the instrument and the droplet of water was then deposited on the polymer surface by a specialized microsyringe. The water droplet was observed from the computer screen and the contact angle was calculated. The porosity of the scaffolds was calculated using Image Analyser Software (Olympus Co. Ltd., Tokyo, Japan). The values of 100 different spots were analyzed and averaged [8].

### 2.4. Antimicrobial Activity

Four bacterial strains, which include *Bacillus licheniformis*, *Staphylococcus aureus* ATCC 12600, *Escherichia coli* ATCC 11303 and *Pseudomonas aeruginosa* ATCC 17588, were used. Briefly, the tested bacterial suspensions (1.5 × 10^6^ CFU/mL) were transferred in sterilized nutrient broth. Then, 20 µL of the bacteria suspension (7.5 × 10^5^ CFU/mL) was added to each antimicrobial coated porous scaffold. The incubation is done under suitable conditions for varied time intervals (0, 6, 12 and 24 h). In every 6 h interval, the scaffold with bacteria adhesion was dissolved in 10 mL of distilled water and vortexed. After that, 100 µL of the bacterial suspension was spread on nutrient agar to observe the colonization of bacteria. The percentage of dead cells is calculated relatively to the growth control by determining the number of living cells (CFU/mL) of each scaffold using the agar plate count method. The percentages of inhibition were calculated using following Equation:

(2) $$C\%=\left(Co-Ce\right)\;/\;Co\times 100\%$$

where *C*% is percentage of inhibition, *Ce* is CFU after incubation period and *Co* is initial CFU before incubation period.

### 2.5. Biocompatibility and Cell Proliferation Evaluation

Mouse fibroblast cell culture (L929, ATCC) was cultured in cell culture flasks containing Modified Eagle Medium (MEM) supplemented with 2 mM L-glutamine, 1.5 g/L sodium bicarbonate, 1 mM of sodium pyruvate, 1000 U/mL penicillin-streptomycin and 10% (*v/v*) of bovine calf serum, which were incubated at 37 °C in 5% (*v/v*) CO_2_ for 2–3 days. The various scaffolds fabricated and its positive control (P(3HB-*co*-4HB) without collagen were cut in size (6 mm in diameter) fitting the 96-well flat bottom culture plate and sterilized under UV cross-linker (Spectrolinker™, XL-1000 UV Cross-linker, Westbury, NY, USA) at 1200 µJ/cm^2^ for 30 min [8,9]. The scaffolds were then placed in the 96-well flat bottom culture plate. Suspension of the mouse fibroblast cell lines (L929) [2.5 × 10^4^ cells/mL] were directly cultivated onto the scaffolds and film. The seeded scaffolds and film were incubated in a 5% (*v/v*) CO_2_ incubator at 37 °C for 96 h. The cells viability and proliferation were assayed with MTS[3-(4,5-dimethylthiazol-2-yl)-5-(3-carboxymethoxyphenyl)-2-(4-sulfophenyl)-2H-tetrazolium/PMS (phenazinmethosulfate). MTS and PMS solution were used to evaluate the biocompatibility of the fabricated of scaffolds. Standard curve was plot based on the cell density from the range of 1 × 10^3^ to 5 × 10^5^ cells/mL. The media was used as the positive control and scaffolds without any incorporation of collagen peptide were used as negative control. The absorbance values were plotted against the counted cell numbers, and thus a standard curve was established [9].

### 2.6. Statistical Analysis

The qualitative results were presented as means and standard deviation (s.d). The qualitative data were analyzed using ANOVA and Tukey’s HSD test with SPSS 20.0 software. All *p* values < 0.05 were considered significant. 

## 3. Results and Discussion

### 3.1. Biosynthesis of P(3HB-co-4HB) via Batch Fermentation

The biosynthesis of P(3HB-*co*-4HB) copolymer was carried out using *Cupriavidus malaysianesis* USMAA1020 transformant, which possessed an excess copy of the phaC gene. This cultivation regulated 4HB molar fraction to achieve 95 mol% of P(3HB-*co*-4HB) with PHA content of 78 wt.% and its concentration at 17.3 g/L in 20 L bioreactor, the mixed substrates of 1,6-hexanediol and 1,4-butanediol at 1:5 ratio. The high 4HB monomers are favored for implantable medical products. This was in agreement with the previous study [23], the 1,6-hexanediol and 1,4-butanediol were utilized as carbon sources as 4-hydroxybutyryl-CoA was initially formed and converted to 4-hydroxybutyrate. The copolymer was recovered by the chloroform extraction method and subjected to physical properties. Based on the results obtained, as summarized Table 2, the average molecular weight (MW) of the polymer was 585 kDa while the polydispersity index was in the range 3.2. Besides, the tensile strength of the polymer was recorded at about 23 MPa with the elongation at break around 611%.

### 3.2. Fabrication of SBCC and SCCC Scaffolds

**Figure 1:**
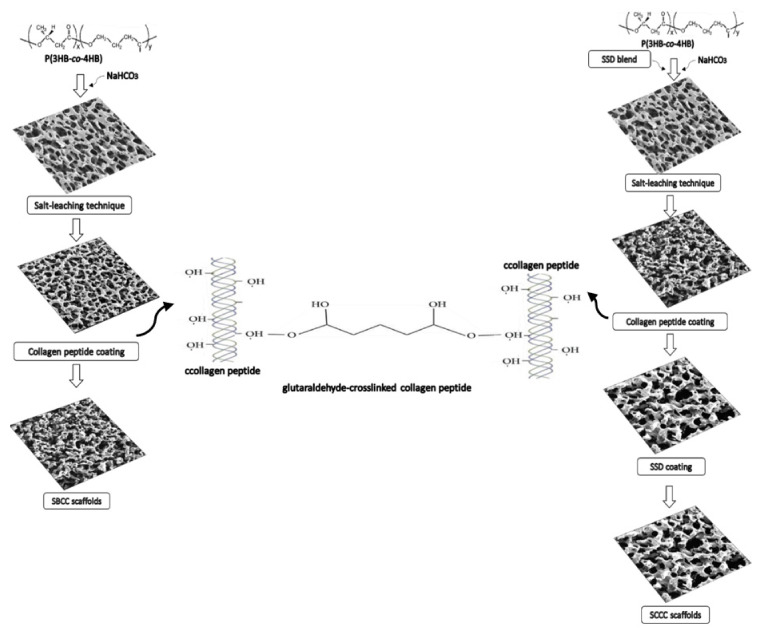
Schematic of fabrication of SBCC and SCCC scaffolds using a combination of salt leaching modification and freeze-drying technique with collagen peptide coating and cross-linking with glutaraldehyde.

In this study a three-dimensional, porous scaffold was successfully engineered with the use of a combination of techniques, namely particle leaching and freeze-drying. Figure 1 shows a schematic of the fabrication of porous antimicrobial SSD-P(3HB-*co*-4HB)-collagen peptide scaffolds termed as SBCC and SCCC scaffolds. The system contained two phases in developing a highly porous, well interconnected pore structure of the scaffold. The first phase involved the particle leaching using NaHCO_3_ (200 µm), followed by the freeze-drying technique. The combination of methods has shown many advantages over other methods as it is easier to control pore structures. This will produce porous scaffolds with open surface pores and interconnected bulk pores which will facilitate cell seeding and homogeneous cell distribution and promote tissue regeneration [26,27]. Despite the homogenous pores’ structures, the surface properties of these polymers are hydrophobic which will possibly inhibit the infiltration of cell suspension into the scaffolds preventing smooth cell seeding.

Therefore, it is crucial to modify the surface characteristic from hydrophobic to hydrophilic to facilitate cell seeding. In this case, the surface of the porous P(3HB-*co*-4HB) scaffolds was coated with hydrophilic collagen peptide to increase the hydrophilicity of the surface, thus improving cell interaction [28,29,30]. In this study, apart from the surface modification of P(3HB-*co*-4HB) scaffold with collagen layer, incorporation of antimicrobial agent, SSD was carried out. This was executed by introducing the SSD either through blending (SBCC) or coating of the scaffolds (SCCC). Fabrication of scaffolds that release the antimicrobial agents or respond to infections is crucial in developing biomaterials in tissue engineering [31,32,33].

**Figure 2:**
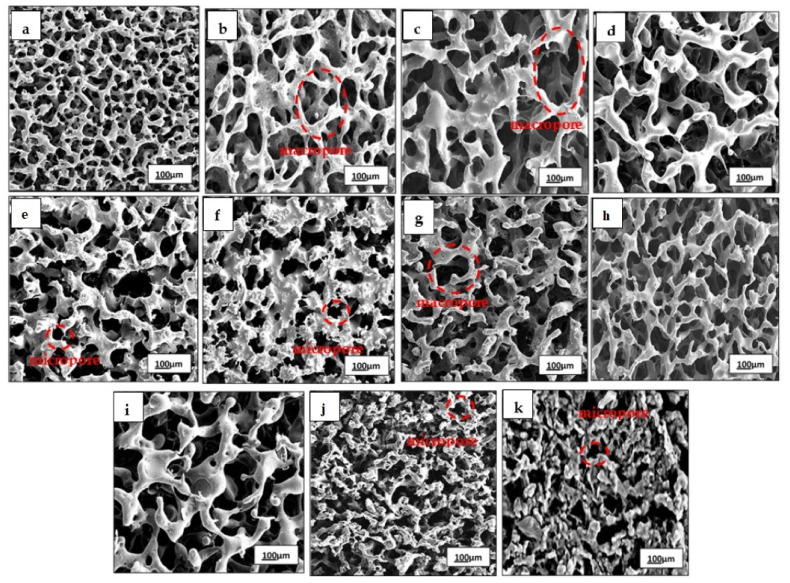
Micrograph of porous structure of (**a**) control-P(3HB-*co*-4HB), (b) SCCC 2.5 wt.%, (**c**) SCCC 5 wt.%, (**d**) SCCC 7.5 wt.%, (**e**) SCCC 10 wt.%, (**f**) SCCC 12.5 wt.%, (**g**) SBCC 2.5 wt.%, (**h**) SBCC 5 wt.%, (**i**) SBCC 7.5 wt.%, (**j**) SBCC 10 wt.% and (**k**) SBCC 12 wt.%.

Besides, surface morphology is crucial in developing biomaterials as this determines the cell-matrix interface interactions. As seen in Figure 2, the SEM micrographs reveal the formation of the three-dimensional interconnected porous structure of SBCC and SCCC scaffolds. Interestingly, the pore sizes observed using SEM were much smaller than the range of porogen sizes (NaHCO_3_) used to create them. This could be attributed to the combination of techniques used, mainly freeze-drying. Hence, combining salt leaching with freeze-drying may enhance pore interconnectivity and assist the formation of homogenous pores ranging from 100 to 200 μm [34]. However, SCCC scaffolds exhibited rougher appearance with less interconnection and possessed numerous macropores as compared to SBCC. Basically, the porous-based connectivity surface is favored to enhance the ECM architecture and provide a larger space to induce cell-material interactions [35,36,37,38,39]. Additionally, both the scaffolds created similar morphology with generally amorphous pores with smooth edge.

**Figure 3:**
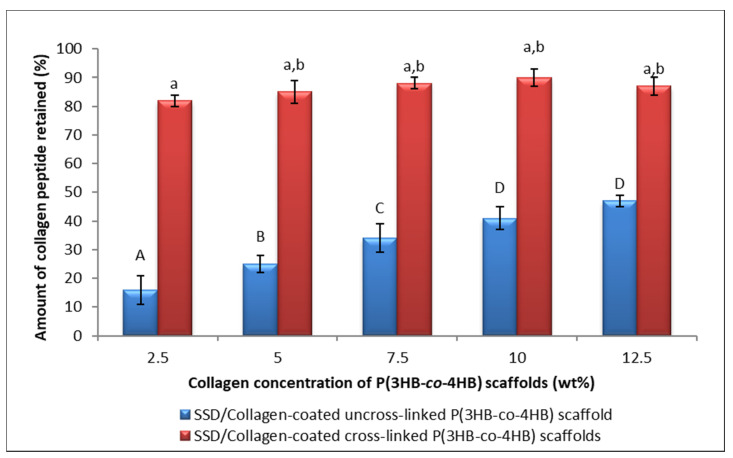
The percentage of collagen retained on cross-linked SCCC and uncross-linked SCCC scaffold. Data represent means ± SD (*n* = 3). Mean data accompanied by different alphabets as of cross-linked SCCC scaffolds (a–b); and uncross-linked SCCC (A–D) indicates significant difference within each respective group (Tukey’s HSD test, *p* < 0.05).

The fabricated scaffolds differed in terms of their construction. SSD coated onto the porous SCCC scaffolds showed solubility in aqueous medium in contrast to the impregnated collagen in SBCC. Hence, the collagen peptide coated P(3HB-*co*-4HB) porous scaffold were then cross-linked via GA vapor phase. The dissolution analysis on scaffolds of cross-linking and uncross-linking scaffolds were shown in Figure 3. The percentage of dissolutions was significantly higher for uncross-linked SCCC scaffolds with the amount retained only between 15% and 45%. On the contrary, the crosslinked SSD/collagen peptide-coated P(3HB-*co*-4HB) scaffold exhibited collagen retain percentage from up to 80% to 90%. This demonstrates that cross-linking with GA enhanced the scaffolds resistance to dissolution. After GA vapor cross-linking, the membranes became visibly yellowish and shrunk dimensionally. The aldimine linkages (CH=N) between the free amine groups of protein and GA attributes to the color change, whereas the covalent bond formed between the aldehyde groups of GA caused shrinkage [40,41]. The aldimine linkage was a reflection of Schiff base reaction, whereby the carbon in the aldehyde group of GA was attacked by nucleophilic nitrogen in the amino group of collagen peptides, and hence replaced the oxygen in the aldehyde group and eliminated water molecule [30]. Nonetheless, GA cross-linking with vapor phase methodology showed low or no detectable cytotoxic effects [25,42]. As described by Teixera et al. (2021), this will enable a sustainable approach in achieving green methodology and the lowest environment impact possible at all stages of fabrication for biomedical application [43].

### 3.3. Functional Group Identification Using FTIR Analysis

**Figure 4:**
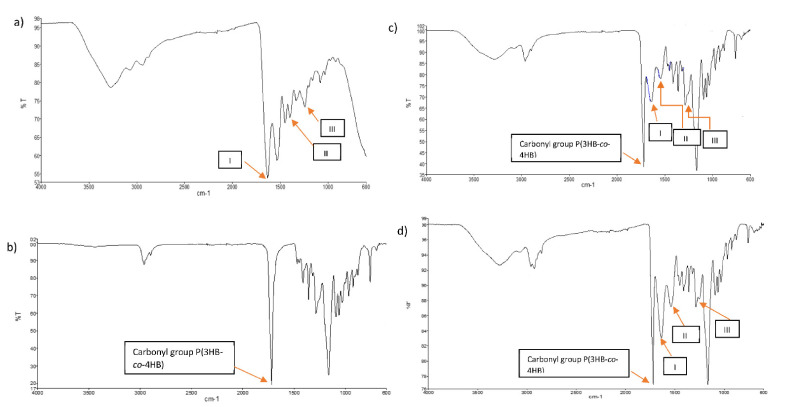
FTIR spectra of scaffolds (**a**) collagen, (**b**) P(3HB-*co*-4HB), (**c**) SBCC, and (**d**) SCCC. Arrows I, II, III indicate amide I, amide II and amide III, respectively.

FTIR analysis shown in Figure 4 was carried as an evidential analysis to determine and analyze the characteristic bands that correlate to functional groups of the fabricated scaffolds. The FTIR spectrum for collagen (a) showed symmetric and unsymmetric stretching of the primary amine (NH_2_) bands at 3275 cm^−1^ and 3150 cm^−1^, respectively. The hydroxyl (OH) from carboxylic acid portion also is expected to be overlapped with the symmetric amine at 3275 cm^−1^. The moderate peaks at 2937 cm^−1^ represent CH_3_ (bend) and CH_2_ (stretch) of the alkanes’ substructure. A strong band at 1633 cm^−1^ represents (C=O) from the amide moiety. Another strong peak can be seen at 1531 cm^−1^ and represents NH_2_ bending [44,45,46,47,48]. The peaks of the (C=C) bands of the aromatic portion also can be clearly observed between the peaks of 1531 cm^−1^ to 1449 cm^−1^. In addition, a moderate peak at 920.89 cm^−1^ would represent a C-H (out-of-plane) band from the aromatics.

In the case of P(3HB-*co*-4HB) polymer (b), moderate peaks at 2963 cm^−1^ and 2899 cm^1^ represent CH_3_ (bend), CH_2_ (stretch) and CH of the alkanes’ substructure. A strong band at 1633 cm^−1^ represents (C=O) and another strong band at 1161 cm^−1^ exhibits the (C-O) band [8,49].

Comparatively, the FTIR spectra of SBCC (c) and SCCC (d) are rather comparable to each other as they exhibit all the expected bands and peaks of the designated collagen, P(3HB-*co*-4HB) polymer and pure SSD. The major characteristic absorption peaks in both FTIR spectra of SBCC (c) and SCCC (d) ca. 3283, 3150, 2900, 1719, 1630, 1540, 1450 and 1164 cm^−1^. The absorption peak at 3283 and 3150 cm^−1^ are assigned to NH_2_ symmetric and asymmetric stretching, respectively. A distinctive peak at 2900 cm^−1^ represents CH_3_ (bend) and CH_2_ (stretch) of the alkanes’ substructure. Whilst the strong peak at 1719 cm^−1^ represents (C=O) peak. The absorption peak at 1630 cm^−1^ corresponds to NH_2_ bending. The peaks at 1540 cm^−1^, 1450 cm^−1^ belong to the peaks of the (C=C) bands of the aromatic portion. The peaks of asymmetric stretching vibration of (SO_2_) group cannot be resolved in these spectra as the band of (C-O) can be dominantly seen in this fingerprint region at 1164 cm^−1^ [50].

It was observed that the prominent characteristic peaks of SBCC (c) and SCCC (d) with a few bands shift in comparison to each other with the dominant characteristics are from the P(3HB-*co*-4HB) polymer (b) which are indicative of the reservation of the chemical aspect of these blends production. It can be concluded that the SSD did not engage with its active groups in any chemical interaction with any of the components of SBCC (c) and SCCC (d) built up. From the FTIR spectra of the two, there is also no evidence of electrostatic interaction nor chemical reaction have taken place between all the materials that made up the blend due to very little shift of all the vibrational wavenumbers (i.e., less than 5 cm^−1^) throughout the major bands of interest.

### 3.4. Porosity Analysis

**Figure 5:**
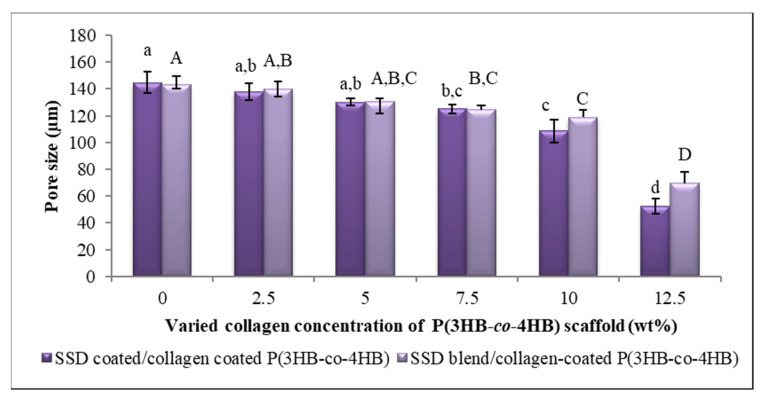
Porosity analysis of SSD coated/collagen coated P(3HB-*co*-4HB); SCCC and SSD blend/collagen coated P(3HB-*co*-4HB); SBCC scaffolds. Data represent means ± SD (*n* = 3). Mean data accompanied by different alphabets as of SCCC scaffolds (a–d) and SBCC scaffolds (A–D) indicates significant difference within each respective group (Tukey’s HSD test, *p* < 0.05).

The pores in scaffolds are imperative as they provide an ideal framework for cells to bind, proliferate and form extracellular matrix. As such, here the porosity was determined with six different collagen concentrations of the scaffolds. The fabricated scaffolds exhibited a gradual drop of the pore size from 145 to 53 µm with increasing collagen concentrations (Figure 5). Similarly, the porosity of the SBCC declined by 50% from the control scaffold. This decrease in porosity could have been due to the larger collagen layer deposits on the surface of scaffolds [51,52,53,54,55]. Based on various studies, pore sizes above 100 µm are ideal for cell infiltration and migration. Interestingly, 10 wt.% scaffolds resulted in a desirable pore size despite the higher concentration of collagen peptide. In developing biomaterial, pore structures of scaffolds play a crucial role in facilitating cell seeding, cell penetration and distribution in the scaffolds. Thus, the adhesion of cells and formation of new tissues and organs occurs [56,57,58]. It is emphasized that an ideal scaffold depends on biomaterial source, fabrication technique and the pore geometry. As such, it is vital to develop a scaffold with specific porosity properties for potential application in tissue engineering and regenerative medicine [59,60].

### 3.5. Hydrophilicity of Fabricated Scaffolds

The hydrophilicity of the SCCC and SBCC scaffolds was determined using water contact angle analysis (Table 3). The graph clearly showed a decline in the contact angle as the concentration of collagen peptide increases, thus indicating the increase of hydrophilicity. Ideally, a contact angle of less than 90° indicates that the surface is wet-prone, hence being categorized as a hydrophilic surface [61,62,63]. Whole wetting was observed with the water droplet becoming a flat puddle with 0° contact angle on SBCC and SCCC with 10 wt.% and 12.5 wt.% collagen peptides. Additionally, the collagen peptide coating enhanced the surface wettability of sample scaffolds. The significant hydrophilicity enhancing effect of collagen peptide could be associated with the amino groups in collagen [64,65].

**Figure 6:**
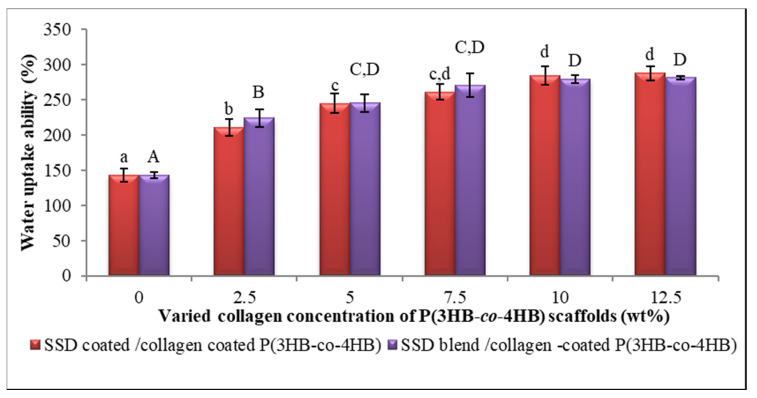
Water uptake analysis of SSD coated/collagen coated P(3HB-*co*-4HB); SCCC and SSD blend/collagen coated P(3HB-*co*-4HB); SBCC scaffolds. Data represent means ± SD (*n* = 3). Mean data accompanied by different alphabets as of SCCC scaffolds (a–d) and SBCC scaffolds (A–D) indicates significant difference within each respective group (Tukey’s HSD test, *p* < 0.05).

The wettability analysis of different sample collagen concentrations is demonstrated in Figure 6. A steady rise of water uptake percentage with the increment of collagen peptide concentrations can be observed. Water uptake ability elucidates the hydrophilicity of fabricated scaffolds which will increase the efficiency of absorption of essential supplements required for cell attachment. Overall, the collagen peptide coated P(3HB-*co*-4HB) scaffold absorbed a larger amount of water, exceeding 100% (*v/v*) of the total volume of the scaffold even at the low concentrations of collagen peptide (2.5 wt.%). As anticipated, the results pointed out that the hydrophilicity of both SCCC and SBCC scaffolds have similar water uptake ability. The water uptake ability properties of scaffolds are crucial in order to enhance the proliferation of a cell. The optimal design of a scaffold strongly depends on both materials and the surface treatment in modulating cell seeding and proliferation [60,66].

### 3.6. Evaluation of Cell Proliferation of Fibroblast Cells on Scaffolds

**Figure 7:**
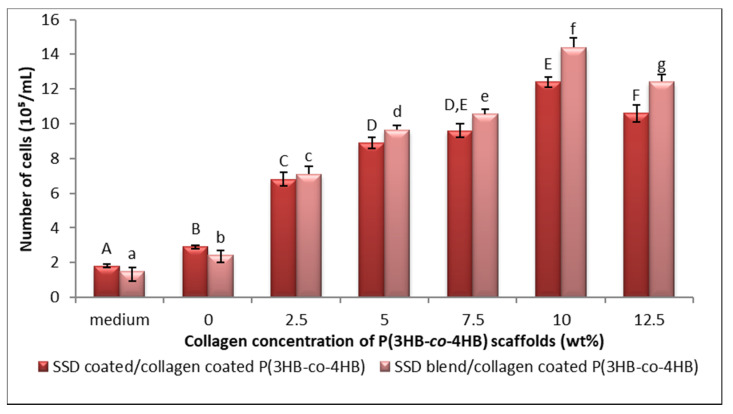
Proliferation of L929 cells on the SSD coated/collagen coated P(3HB-*co*-4HB); SCCC and SSD blend/collagen coated P(3HB-*co*-4HB); SBCC scaffolds. Data represent means ± SD (*n* = 3). Mean data accompanied by different alphabets as of SCCC scaffolds (A–F) and SBCC scaffolds (a–g) indicates significant difference within each respective group (Tukey’s HSD test, *p* < 0.05).

**Figure 8:**
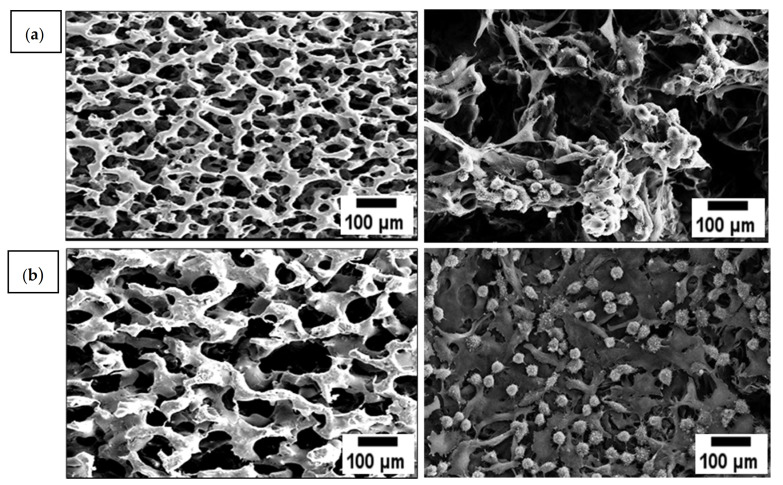
Micrograph of proliferation of L929 cells on (**a**) control-P(3HB-*co*-4HB), SCCC scaffolds (**b**) SCCC 10 wt.%. Data represent means ± SD (*n* = 5).

In general, a functional scaffold requires the ability to support attachment and promote proliferation of cultured cells [67]. In line with it, the L929 fibroblasts cells behavior towards SCCC and SBCC scaffolds with different collagen concentrations was investigated as shown in Figure 7. Cells adhered well with progressive growth and by day three, the scaffold surfaces supported high cell density. The cell proliferation was spotted to increase significantly on scaffold coated with 2.5 wt.% until it reaches 10 wt.% as compared to the collagen free scaffold. However, the number of fibroblast cells decreased (10.6 × 10^5^ cells/mL) at the highest collagen peptide concentration (12.5 wt.%). This may be attributed to the reduction of pore size, which caused less pore accessibility and proliferation [8,30,68,69]. In the current study, 10 wt.% collagen coated scaffold with pore size around 108.6 ± 8.7 µm demonstrated highest proliferation rate (12.4 × 10^5^ cells/ mL), as shown in Figure 8, in comparison to control, as well as 2.5 wt.%, 5 wt.% and 7.5 wt.% collagen coated scaffolds. In short, scaffolds fabricated using combined techniques displayed the highest cell proliferation. These findings clearly implied the enhancement of cell proliferation attributes to the effects of collagen on cell viability. In short, these findings clearly demonstrated the process of incorporating collagen layer on the scaffold is an efficient way to initiate cell attachment and supports cell growth [63,64].

### 3.7. Antimicrobial Analysis of SCCC and SBCC Scaffolds

**Figure 9:**
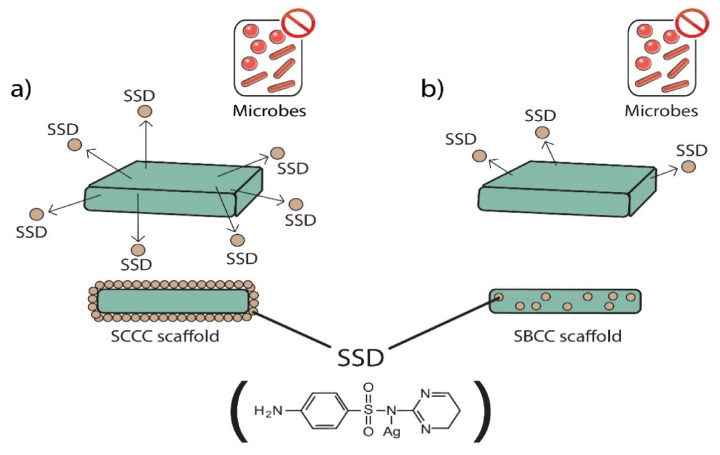
Schematic represents the releasing rate of silver ion from (**a**) SCCC scaffolds which rapidly release SSD and (**b**) the slow release of silver ion impregnated in the SBCC scaffolds.

Antimicrobial analysis was carried out using the colonization test as summarised in Table 4. Antimicrobial substance, silver sulfadiazine (SSD), was incorporated in the scaffolds. Silver compounds, especially (SSD), has been widely used as an antibacterial agent in various biomedical applications [69,70]. Based on the results obtained, both SCCC and SBCC scaffolds revealed desirable antimicrobial effects. However, SBCC scaffolds required 48 h to inhibit certain pathogenic microorganisms which was due to the elution of silver sulfurdiazine impregnated with SSD possessed, whereby Ag ions were physically entrapped in the scaffolds where controlled release of antimicrobial agent occurred [70]. Meanwhile, the results revealed that in SCCC with scaffolds, the silver ion was continuously released directly leading to almost 100% inhibition for most of the microorganism within 12 h. Both scaffolds showed different functionality according to the releasing rate of silver ion. The schematic of the antimicrobial release of both the scaffolds is illustrated in Figure 9. The SCCC scaffolds, which rapidly release SSD, are thus appropriate for further work towards dermal application, especially skin damage to the epidermis and the upper dermis that can be regenerated spontaneously and healed in relatively shorter periods [71,72,73,74]. On the condition of chronic wounds, such as diabetic ulcers, long-term release of antimicrobials is highly suggested since regeneration occurs at the edges of injuries [75]. Therefore, the SBCC scaffold can be beneficial for such cases. The antimicrobial effect of SBCC scaffold is effective by the significantly prolonged release of silver ion, which continues to kill microbes after the release system is exhausted. The release of silver ions is accompanied by the contact killing of the layer that contains silver ion gradually released by diffusion and scaffold degradation [69]. Furthermore, according to Heo and coworkers [73], silver sulfadiazine binds with microbial DNA and releases the sulfonamide, interfering with the intermediary metabolic pathway [76].

## 4. Conclusions

In this study, we demonstrated that a combination of a simple and green approach to fabricate collagen and SSD incorporated P(3HB-*co*-4HB) scaffolds using porogen leaching and freeze-drying techniques. In comparing the SCCC and SBCC scaffolds, both the scaffolds differed in the incorporation of antimicrobial agent. Biomaterial based microbial infections pose serious concerns in the biomedical field. This study focuses on the development of highly efficient potential biomaterials that release the antimicrobial agents. This is in response to the limitations caused by some biomaterials with antimicrobial properties that inhibit microbial infections but slow down the cell seeding and tissue integration. Here, both the SCCC and SBCC scaffolds enhanced cell seeding and proliferation of L929 cells. Nonetheless, SCCC has higher antibacterial efficiency within the first 24 h, whereby the antibiotic is rapidly released as compared to the controlled release of the antimicrobial properties in SBCC scaffolds. Entrapment of SSD in P(3HB-*co*-4HB), as in SBCC, resulted in a reduced burst release of SSD as compared to SCCC. Nonetheless, both the SCCC and SBCC scaffolds could be an excellent candidate to inhibit microbial colonization based on the biomaterial application without causing antibiotic resistance. The study provides evidence and elucidates the surface interface-cell interactions of the modified P(3HB-*co*-4HB) scaffolds and release of the antimicrobial agent from the scaffolds, thus paving the way in developing infection-resistance biomaterials in the biomedical field in the future.

## Figures and Tables

**Table 1:** List of common examples of various antimicrobial scaffolds incorporated with SSD.

Biopolymer/Materials	Fabrication of Scaffolds	Applications	References
Collagen/SSD	Facile blending	Wound dressings	[19]
Collagen/SSD	Electrospinning	Wound healing applications	[15]
Collagen/SSD	Blending with SSD-loaded alginate microspheres	Conventional burn dressings in second-degree burns	[16]
Polycaprolactone (PCL)/SSD	Electrospinning	Antibacterial scaffold	[20]
P(3HB-*co*-4HB)/collagen peptide/SSD	Aminolysis	Potential wound healing	[9]
Polycaprolactone (PCL) and Polyvinyl alcohol (PVA)/SSD	Electrospinning	Antimicrobial wound dressing	[21]
Poly(lactic acid) (PLA)/SSD	Electrospinning, structural reconstruction	Antimicrobial wound dressing	[22]

**Table 2:** Physical and mechanical properties of P(3HB-*co*-4HB).

Copolymer	Tensile Strength	Elongation at Break	Young Modulus	M_w_	M_n_	PDI ^b^
	(MPa) ^a^	(%) ^a^	(Mpa) ^a^	(kDa) ^b^	(kDa) ^b^	
P(3HB-*co*-95 mol% 4HB)	23.2 ± 4	611.8 ± 1	226.6 ± 20	585 ± 8	132 ± 11	3.2 ± 0.5

Values are mean ± SD of three replicates; ^a^ Determined using Gotech Al-3000 tensile Machine; ^b^ Calculated from GPC analysis, M_n_: number-average molecular weight; M_w_: weight average molecular weight; M_w_/M_n_: polydispersity index.

**Table 3:** Water contact angle of scaffolds with various collagen peptide concentration.

Collagen Peptide (wt.%)	Types of Scaffolds	
	Coat/Coat	Blend/Coat
0	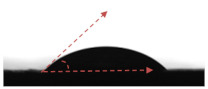 49.9 ± 2.7	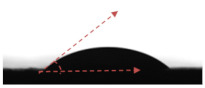 73.3 ± 1.4
2.5	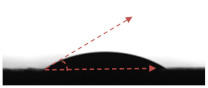 32.5 ± 2.8	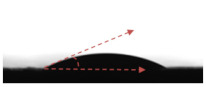 45.2 ± 2.4
5.0	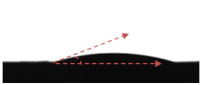 15.3 ± 3.3	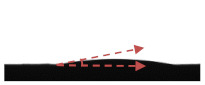 25 ± 5.4
7.5	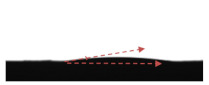 8.58 ± 0.8	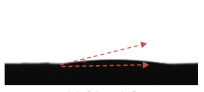 14.81 ± 1.2
10.0	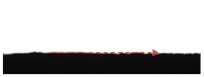 0	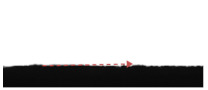 0
12.5	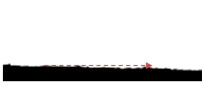 0	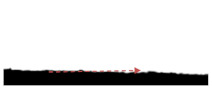 0

**Table 4:** Antimicrobial test of SCCC and SBCC scaffolds against various microorganisms.

Time (h)	Inhibition of Microorganisms (%)							
	6		12		24		48	
	SCCC	SBCC	SCCC	SBCC	SCCC	SBCC	SCCC	SBCC
*Staphylococus aerus* ATCC 12600	65 ± 5	13 ± 1	85 ± 3	36 ± 5	100 ± 0	83 ± 8	NA	100 ± 0
*Escherichia coli* ATCC 11303	79 ± 8	34 ± 5	100 ± 0	51 ± 9	100 ± 0	92 ± 6	NA	100 ± 0
*Pseudomonas aeruginosa* ATCC 17588	85 ± 7	43 ± 9	100 ± 0	45 ± 5	100 ± 0	87 ± 12	NA	100 ± 0
*Bacillus licheniformis*	98 ± 2	65 ± 10	100 ± 0	95 ± 5	100 ± 0	100 ± 0	NA	100 ± 0
*Candida albicans*	93 ± 7	33 ± 6	100 ± 0	71 ± 10	100 ± 0	94 ± 6	NA	100 ± 0

Values are mean ± SD of three replicates; NA denotes not applicable.

## Data Availability

The data presented in this study is openly available.
